# A Clinical Treatise on the Diseases of the Nervous System

**Published:** 1879-11

**Authors:** 


### BIBLIOGRAPHICAL.

A Clinical Treatise on the Diseases of the Nervous System.—By M. Rosenthal, Professor of Diseases of the Nervous System, of Vienna, with a preface by Prof. Charcot, translated from the author's revised and enlarged edition by L. Putzell, M.D., visiting physician for nervous diseases, Randall's Island Hospital, etc., etc. Vol. II. Wm. Wood & Co., New York, 1879.

The merit of the first volume is well sustained in this the second. For a treatise on the diseases of the nervous system, there is no work better arranged or more scientifically executed. The author is identified with the more advanced discoveries and researches in this most difficult field of medical science, and we may safely assert that no other book will give more benefit or information on nervous diseases.

